# In response to ‘Medical Education Research: is participation fair?’

**DOI:** 10.1007/s40037-015-0188-6

**Published:** 2015-05-30

**Authors:** W. E. Sjoukje van den Broek, Roel H. P. Wouters, J. J. M. Hans van Delden

**Affiliations:** 1 Netherlands Association for Medical Education (NVMO) Ethical Review Board, Utrecht, The Netherlands; 2 University Medical Center Utrecht Medical School, Room HB 4.05, PO Box 85500, 3508 GA Utrecht, The Netherlands; 3 Julius Center for Health Sciences and Primary Care, University Medical Center Utrecht, Utrecht, The Netherlands

We would like to respond to the eye-opener by Walsh [1], in which he comments on the heavy reliance on undergraduate students as participants in medical education research. Walsh mentions two key threats to the fairness and validity of research with these participants: (1) Medical students might feel pressure to participate in research because researchers are, in most cases, faculty/ their tutors, and (2) the overrepresentation of undergraduate students because they are easy to include in research projects.

Concerning the first threat, we think it is important to distinguish between *vulnerability* and *undue influence*. According to the Declaration of Helsinki [2], vulnerable groups and individuals ‘may have an increased likelihood of being wronged or of incurring additional harm.’ In some cases, persons are vulnerable because they are relatively (or absolutely) incapable of protecting their own interests. This may occur when persons have relative or absolute impairments in decisional capacity, education, resources, strength, or other attributes needed to protect their own interests. We consider medical students to be reasonable and autonomous adults, capable of making a wide variety of decisions in life. As such they would not immediately strike us as vulnerable. Undue influence, however, is a relevant concern in educational research because there unmistakably is a power differential between student and staff. It is worth noticing here, however, that undue influence may also be present in other examples of education research. Residents, for example, may also experience pressure from their supervisors to participate in research. In the Netherlands, the NVMO Ethical Review Board pays particular attention to the risk for undue influence for all participants involved [3].

Concerning the second threat, we recognize the problematic overrepresentation of undergraduate students in research mentioned by Walsh. Yet oversight by ethics committees will not be sufficient to address this problem as they simply lack the required oversight and the means to influence research agendas. Instead of calling for oversight we suggest that persons that are invited to participate in research must be selected for scientific reasons and not because they are easy to recruit. In addition researchers should justify to a research ethics committee the exclusion of other groups. Equity indeed requires that the benefits of research be distributed fairly and that no group or class of persons bear more than its fair share of the risks or burdens from research participation.

## Conflict of interest

All authors are board members of the Netherlands Association for Medical Education (NVMO) Ethical Review Board.
